# Measuring the Brain-Gut Axis in Psychological Sciences: A Necessary Challenge

**DOI:** 10.3389/fnint.2019.00073

**Published:** 2020-01-09

**Authors:** Ismael Palacios-García, Francisco J. Parada

**Affiliations:** ^1^Laboratorio de Psicofisiología, Escuela de Psicología, Pontificia Universidad Católica de Chile, Santiago, Chile; ^2^Laboratorio de Neurociencia Cognitiva y Social, Facultad de Psicología, Universidad Diego Portales, Santiago, Chile

**Keywords:** brain, gastrointestinal system, psychology, neuroscience, 4E-cognition, microbiota

## Introduction

The brain/body relationship and interdependence has been one of the most prevalent questions toward understanding psychobiological mechanisms underlying human behavior (Gover, 1996; Thompson and Varela, 2001). Current epistemological stances define brains as dynamic, complex, and self-organized systems (Cosmelli and Thompson, 2011), tightly coupled, and integrated with the rest of the body, establishing bidirectional communication axes (Thayer and Lane, 2000; Craig, 2002). The paradigmatic turn can be evidenced in an increment in scientific research considering both brain and bodily signals, such as the heart (Pollatos et al., 2007b; Villena-Gonzalez et al., 2017), respiration (Yuan et al., 2013; Ahani et al., 2014), gastrointestinal (Richter et al., 2017; Rebollo et al., 2018), and muscular dynamics (Boonstra et al., 2009, 2015; Kerkman et al., 2018). Recent evidence has furthermore revealed the many ways in which psychological processes influence the body, and vice-versa, with behavioral and health implications (Pollatos et al., 2007a; Mattson, 2015; Babo-Rebelo et al., 2016; Azzalini et al., 2019).

These ideas are products of continuous epistemological growth, already present at the end of behaviorism and the early days of the cognitive revolution. From Bartlett in the UK to Dewey in the USA to Luria in Moscow, many scientists had seen mind and brain as a whole with the body (Rossi et al., 2019). Like them, many other revolutionaries -whose work was unaffected by behaviorism- pushed forward the idea of a mind without the need for *manipulation of abstract symbols and representations*. However, the mainstream epistemological stance in Psychological and Cognitive Sciences still pursues the anthropogenic representational and computational capacities of the mind (Frégnac, 2017; Hari, 2017; Jonas and Kording, 2017).

## Body Signals Influence Mood and Behavior

Research agendas including brain/body measurements are sustained in part by the fact that there are intrinsic cognitive mechanisms, related to body awareness and sense of self, integrating and monitoring visceral information; a process known as *interoception* (Craig, 2002; Slonim, 2014; Quadt et al., 2018). Interoception is a global concept encompassing a plethora of processes: neuro/humoral body-to-brain signals, neurocognitive dynamics associated to the integration of those signals, the influence of those dynamics on extended brain/body functional networks, and the associated unfolding of metacognitive processes (Valk et al., 2016; Quadt et al., 2018). High interoception has been associated with increased emotional regulation (Füstös et al., 2012) and decreased alexithymia (Herbert et al., 2011), depression (Avery et al., 2014), and anxiety (Garfinkel et al., 2016). Hence, it has been suggested that accurate sensing of visceral information and body awareness is a critical factor for psychological and emotional regulation, well-being (Hanley et al., 2017), and the basis for an integrated experience of the *self* (Christoff et al., 2011). The “Neural Subjective frame” hypothesis (Park and Tallon-Baudry, 2014) integrates evidence suggesting body signals can, non-consciously, modulate other cognitive processes like self-processing (Babo-Rebelo et al., 2016) and perception (Park et al., 2014). This hypothesis suggests that part of the emotional experience and perception is sustained by implicit and continuous brain monitoring of the internal organs of the body, such as the heart. These process would depend on autonomic signals mediated by the vagus nerve (Slonim, 2014).

It seems plausible that physical and mental well-being might depend on states emerging from implicit and explicit information associated with bodily signals (Critchley, 2005; Farb et al., 2012). Interestingly, one of the greatest sources of body information comes from the gastro-intestinal system (Park and Tallon-Baudry, 2014; Azzalini et al., 2019). This latter point has been an important focus of recent research and increasing evidence identifies gut microbiota as playing a functional role on cognition and emotion (Cryan and Dinan, 2012; Allen et al., 2017; Sarkar et al., 2018).

## Gut Microbiota Influence Behavior

The relationship between nervous and gastrointestinal systems is an example of psychobiological integration with direct impact on health, well-being (Grenham et al., 2011; Mayer et al., 2014; Carabotti et al., 2015; Fukui et al., 2018), and psychological states such as stress and anxiety (Mackos et al., 2016; Provensi et al., 2019). In fact, exposure to social stressors changes microbiota composition (Bailey et al., 2011) and diversity (Partrick et al., 2018), in a process that may also influence the immune function (Gur and Bailey, 2016). Interestingly, the treatment with bacteria of the *Bifidobacterium* and *Lactobacillus* genus confer resilience against effects of stress (Bharwani et al., 2017; Yang et al., 2017). It is worth mentioning that most of the evidence have been acquired using animal models. Hence, understanding the bidirectional role of psychological processes over microbiota in humans is still lacking. Microbiota would impact behavior via bottom-up pathways, positioning it as a factor to consider in studies attempting the understanding of well-being (O'mahony et al., 2009; Dinan and Cryan, 2012; Dinan et al., 2013). Moreover, increasing evidence has posited microbiota as relevant in the context of autism (Mulle et al., 2013; Sgritta et al., 2019), schizophrenia (Severance et al., 2016), multiple sclerosis (Jangi et al., 2016), bipolar disorder (Evans et al., 2017), irritable bowel disease (Jeffery et al., 2012; Kennedy et al., 2014), obesity (Gomes et al., 2018), neurodegenerative disorders (Boehme et al., 2019), and depression (Naseribafrouei et al., 2014; Jiang et al., 2015; Aizawa et al., 2016; Kelly et al., 2016; Heym et al., 2019). The mechanisms through which microbiota exert its effects over behavior include neural pathways via the vagus nerve, regulation of the stress response, production of short chain fatty acids after fiber fermentation, amino acids metabolism and control of immune function, among others (Cryan and Dinan, 2012; Ma and Ma, 2019). The crosstalk between microbiota and immune cells is particularly relevant in therapeutic contexts, as a tight and complex relationship between dietary composition (amino acids) and inflammatory regulation by microbiota-dependent metabolic processes exists (Ma and Ma, 2019; Ma et al., 2019). Thus, positioning diet as a relevant therapeutic alternative for inflammatory-related conditions affecting brain and gut (Kiecolt-Glaser et al., 2017; Valdes et al., 2018).

Hence, growing evidence posits the gastrointestinal system in general -and the microbiota in particular- as a fundamental regulator of nervous system functioning (Agustí et al., 2018; Davidson et al., 2018) with clear neurobiological mechanisms (Cryan and Dinan, 2012; Ma and Ma, 2019) and potential impact on health and behavior. Our comprehension of cognitive and affective processes might depend on understanding the composition, diversity, and physiology of this ecosystem of microorganisms. In the fledgling field of gut-brain axis research, a plethora of novel questions emerge, some of them focused on understanding the particular role of specific bacterial strains on cognition, behavior, and overall brain function.

## The Use of Probiotics as a Behavioral Regulator

Clinical population studies have pointed at the role of specific bacterial strains in brain function and their use as probiotics have adopted the name of *psychobiotics* (Dinan et al., 2013). For instance, patients diagnosed with depression present a decreased population of *Bifidobacterium, Lactobacillus* bacteria, and *Faecalbacterium* (Aizawa et al., 2016). Accordingly, psychobiotics with different combinations of strains have been used to assess their effects over depression symptoms in healthy participants and clinical population (Pirbaglou et al., 2016). For instance, depressive symptoms are diminished after 30 days of probiotic formulation with *Lactobacillus helveticus* and *Bifidobacterium longum* (Messaoudi et al., 2011). Likewise, patients diagnosed with irritable bowel syndrome scoring high in depression were treated with *Bifidobacterium longum* strain probiotics for 6 weeks, resulting in a significant decrease of subjective levels of depression (Pinto-Sanchez et al., 2017). Psychobiotics have also been used in the context of social/cognitive processes such as assessing attention (Chung et al., 2014), emotional processing (Tillisch et al., 2013) and stress (Allen et al., 2016). Additionally, it has been also shown that brain signatures -under MRI setup- of healthy participants during an emotional memory and decision-making tasks are sensitive to 4-weeks of psychobiotic administration (Bagga et al., 2018). This effect was also accompanied with behavioral, self-reported and microbiota changes, suggesting that gut dynamics affect cognitive processes and the associated brain correlates.

The aforementioned results could be explained, in part, through bidirectional neural circuits established between the central nervous system, the enteric system, and the vagus nerve (Forsythe et al., 2014). This hypothesis has been tested using animal models in which anxiolytic and antidepressant effects induced by *Bifidobacterium longum* strains probiotics are blocked after the section of the vagus nerve (Bercik et al., 2011; Bravo et al., 2011). Hence, vagal afferents are necessary for any cognitive, affective, and behavioral effects produced by these microorganisms (Han et al., 2018). Additionally, a recent study identified a type of enteric sensory cell that, by means of a single synapse with neurons of the vagus nerve, propagates nutrient information from gut to brain in the order of milliseconds (Kaelberer et al., 2018). This communication channel may also include information from microbiota-dependent immune dynamics of the gut mucosa (Ma et al., 2018). Furthermore, low-frequency gastro-intestinal oscillations (0.05 Hz) and cortical alpha rhythms (8–10 Hz) coupling has been described (Richter et al., 2017), indicating that the cross-talk between gut-microbiota and brain may be faster and more direct than previously thought. Complementarily, a gastric network was described during resting state involving connectivity between gastric oscillations and brain regions related to the generation of alpha rhythms and visual, somatosensory, and motor internal body representations (Rebollo et al., 2018). Those neural oscillatory networks could shed light on a possible physiological mechanism by means of which the microbiota communicates with the brain, exerting effects on mental processes in a fast and direct way (Komanduri et al., 2019). Nevertheless, this is a fledgling field and much research is still needed.

## Discussion and Outstanding Questions

**Figure 1:**
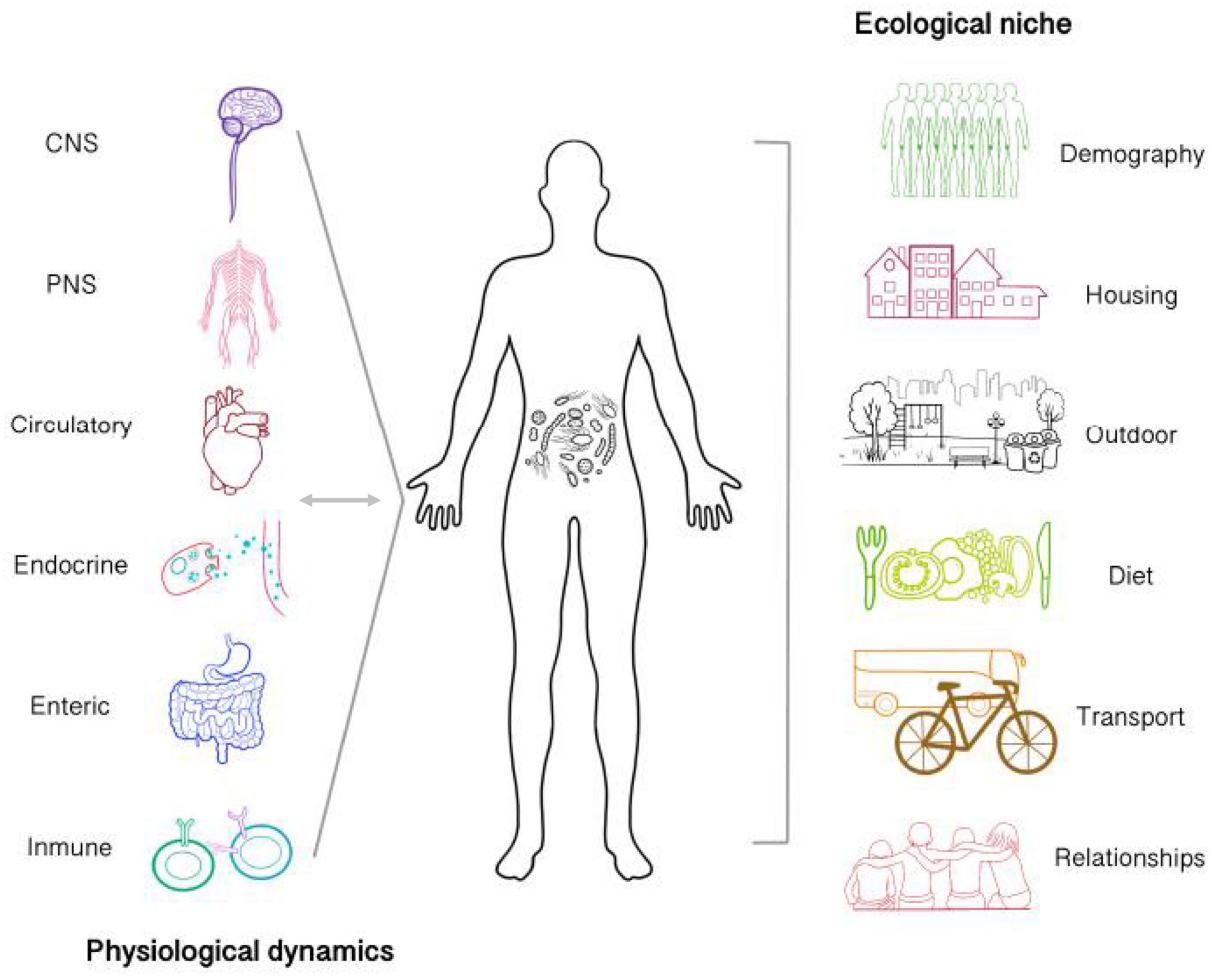
Microbiota establishes bi-directional relationships with physiological processes of the body and is affected by the ecological niche of which an agent participates. Gut microbiota interacts with other systems via neural and humoral pathways (Central, enteric and peripheral nervous system, immunoendocrine pathways, etc.). Possible communication mechanisms involve the regulation of neurotransmitter metabolism, gut permeability, nutrient processing and absorption, inflammatory cytokines release, the stress response, etc. Therefore, internal microbiota dynamics are actively affecting body dynamics in a process which in turn is also affecting microbiota. Microbiota in not only affected by internal processes but also by the agent's ecological niche. The features of the environment at different levels might be limited for the colonization and establishment of specific microbiological communities. Some environmental features which could be relevant includes the amount of people, the presence of the urban green space, urban hygiene, etc. Additionally, the ways by which the subject interact with it such as transportation, diet, and interpersonal relationships would also provide relevant information to take into account at the moment of carrying out interdisciplinary microbiota research.

When considering dynamics internal to the organism (Figure 1), understanding how the brain-gut-microbiota establishes bidirectional relationship offers new perspectives that will greatly advance our comprehension of phenomena studied by psychology, neuroscience, and psychiatry (Tillisch et al., 2013; Dinan and Cryan, 2017). Given the increment of brain-gut-microbiota research, some important research questions have emerged. First, the physiological mechanisms underlying its relation to other body systems and health, in general, remains unknown in humans, resulting in limited clinical applicability (Schmidt et al., 2018; Zmora et al., 2019). Second, the establishment of microbiota communities begins early in development, even before birth. It has been suggested that pre- and postnatal experiences affect microbiota composition, shaping the immune system's function, ultimately leading to increased risk of disease (Tamburini et al., 2016; Francis and Dominguez-Bello, 2019). However, most countries are still far away from including the microbiota as a relevant factor for public policymaking. Finally, public opinion on probiotic products (from Kefir to Kombucha to laboratory formulas) has become favorable. However, recent evidence suggests that intake of generic probiotic formula as a therapeutic alternative should be carefully considered, as gut mucosal colonization presents person-specific resistance to probiotics (Zmora et al., 2018). Therefore, consuming such products as means of life-quality improvement and disease prevention might barely work. In contrast, it seems probiotic formulas will have to contain specific bacterial strains personalized for particular individuals, according to each person's diet (Oriach et al., 2016). Furthermore taking into account other physiological parameters relevant for the host-microbiota interplay, such as nutrients availability and water absorption (Arnoldini et al., 2018).

Considering the ecological niche of the organism presents a major challenge for microbiota research (Figure 1). Given the available evidence of living environment in mental health [i.e., housing quality, indoor/outdoor noise, occupant density, etc. (Evans, 2003)], the connection between the community of microorganisms residing in built environment and well-being remains unknown (Kembel et al., 2014; Relman et al., 2014). These microorganisms, grouped within the fledgling literature of the *microbiome of the built environment* (MoBE), have co-evolved with the mammalian immune system. Hence, there is a good reason to believe that the increment of chronic inflammatory disorders and others such as Alzheimer in industrialized countries might relate to reduced or increased exposure to certain microbial communities (Raison et al., 2010; Fox et al., 2013). Furthermore, research on the impact of MoBE in the development and dynamics of the community and/or person-specific microbiota is needed (Huttenhower et al., 2012; Hoisington et al., 2015; Lax et al., 2015). Thus, providing opportunities for specific and strategic MoBE manipulation that will ultimately regulate microbial diversity in order to reach positive outcomes.

## Concluding Remarks

Cognitive process and associated states such as well-being are embodied, in a process of phylogenetic and ontogenic interdependencies, encompassing an organism's both internal and external environments. Diurnal mammals' physiology has been enslaved by the day/night cycle, *imposed* to planet Earth from the cosmos (Parada and Rossi, 2018). Mammals' physiology is furthermore entangled to the micro-dynamics of small organisms, *imposed* onto the body through the development of a symbiotic relationship unfolding throughout ontogeny and phylogeny. Therefore, adequate scientific study of human behavior will include as many levels as possible: socio-cultural, psychological, microbiological, etc. (Parada and Rossi, 2018). The brain-gut-microbiota topic represents a fascinating opportunity to expand our knowledge about cognition, mental health, and life in general. It is important to frame this research topic from multiple perspectives including biological/medical sciences, public policy, architecture, urbanism, and psychology. Furthermore, recent philosophical and epistemological advances, under the 4E-cognition framework (Newen et al., 2018), will help the integration of evidence, providing new insights and novel hypotheses.

## Author Contributions

IP-G and FP conceptualized the present work and wrote the current version for publication.

### Conflict of Interest

The authors declare that the research was conducted in the absence of any commercial or financial relationships that could be construed as a potential conflict of interest.
